# An Update to Hallmarks of Cancer

**DOI:** 10.7759/cureus.24803

**Published:** 2022-05-07

**Authors:** Swapna Ravi, Antonio M Alencar, Jemma Arakelyan, Weihao Xu, Roberta Stauber, Cheng-Chi I Wang, Ruzanna Papyan, Narine Ghazaryan, Rosalina M Pereira

**Affiliations:** 1 Department of Medicine, St. Luke's Hospital, Duluth, USA; 2 Department of Medical Oncology, Hospital Universitário da Universidade Federal do Maranhão, Hospital São Domingos, São Luís, BRA; 3 Department of Oncology/Solid Tumors, Yerevan State Medical University, Hematology Center After Prof. R. Yeolyan, Yerevan, ARM; 4 Department of Business Development, Harbour BioMed, Boston, USA; 5 Department of Oncology, Universidade do Estado do Rio de Janeiro, Rio de Janeiro, BRA; 6 Department of Research and Development, Beltie Bio, Inc, San Diego, USA; 7 Department of Pediatric Oncology and Hematology, Yerevan State Medical University, Pediatric Center and Blood Disorders Center of Armenia, Yerevan, ARM; 8 Department of Molecular Biology, L.A. Orbeli Institute of Physiology National Academy of Sciences, Republic of Armenia (NAS RA) Hematology Center After Prof. R. Yeolyan, Yerevan, ARM; 9 Department of Neurology, University of Texas Southwestern Medical Center, Dallas, USA

**Keywords:** genome instability, macroenvironment, microenvironment, autophagy, tumor, hallmark, cancer

## Abstract

In the last decade, there has been remarkable progress in research toward understanding and refining the hallmarks of cancer. In this review, we propose a new hallmark - “pro-survival autophagy.” The importance of pro-survival autophagy is well established in tumorigenesis, as it is related to multiple steps in cancer progression and vital for some cancers. Autophagy is a potential anti-cancer therapeutic target. For this reason, autophagy is a good candidate as a new hallmark of cancer. We describe two enabling characteristics that play a major role in enabling cells to acquire the hallmarks of cancer - “tumor-promoting microenvironment and macroenvironment” and “cancer epigenetics, genome instability and mutation.” We also discuss the recent updates, therapeutic and prognostic implications of the eight hallmarks of cancer described by Hanahan et al. in 2011. Understanding these hallmarks and enabling characteristics is key not only to developing new ways to treat cancer efficiently but also to exploring options to overcome cancer resistance to treatment.

## Introduction and background

**Figure 1:**
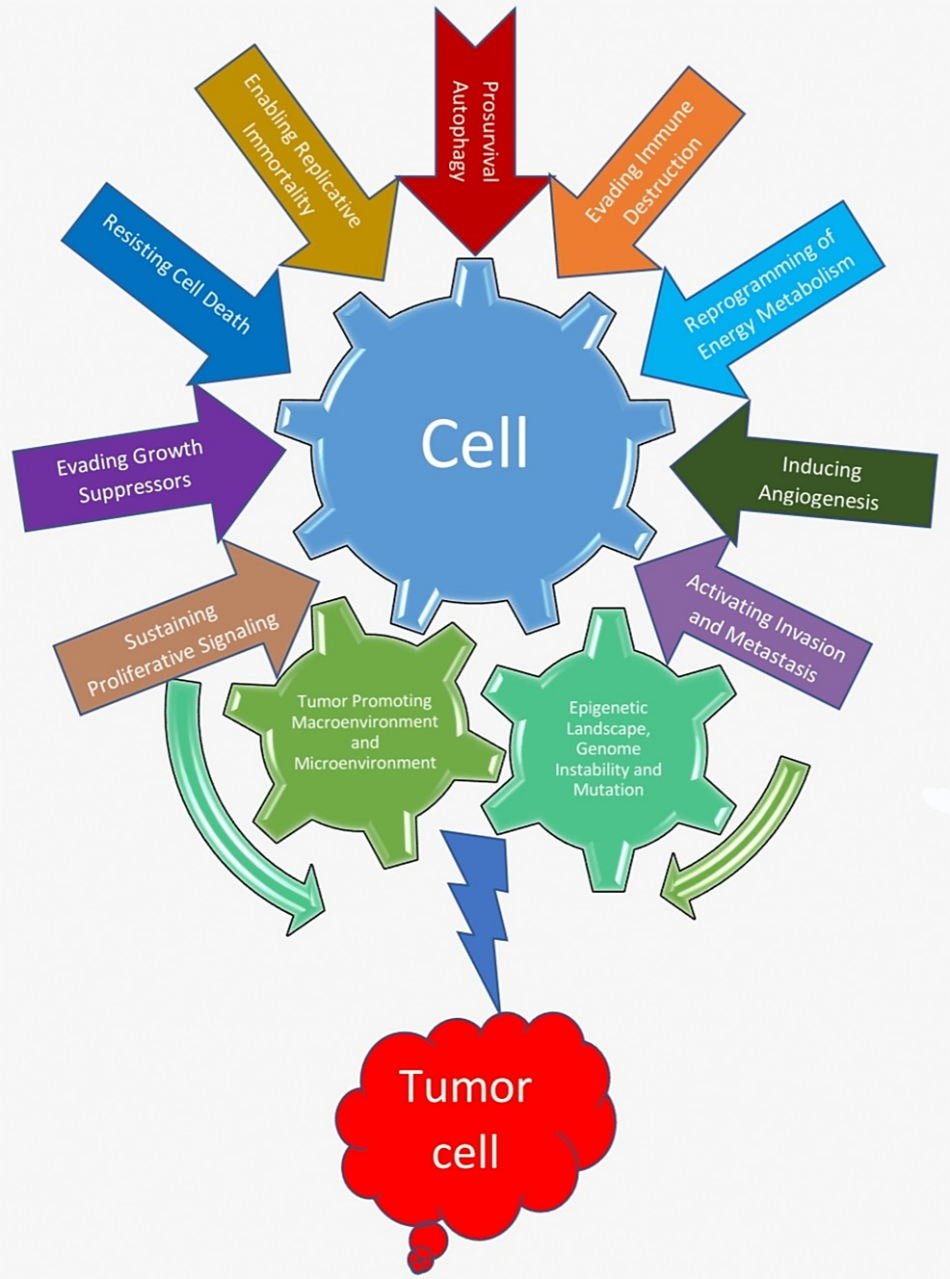
Hallmarks of cancer

The transformation of a normal cell into a neoplasm is a complex process. Hanahan et al. summarized hallmarks of cancer including six core hallmarks, two emerging hallmarks and two enabling characteristics [1]. The bidirectional communication between a cancer cell and tumor microenvironment (TME) and macroenvironment creates a tumor-supportive environment and premetastatic niche, which helps cancer cell to develop, grow and metastasize [2]. In the last decade, there has been tremendous progress in research to understand the hallmarks of cancer. Hanahan et al. emphasized the importance of autophagy, independently or in association with apoptosis, as a barrier that needs to be avoided by cancer cells to reach efficient tumorigenesis, as part of resisting cell death hallmark of cancer [1]. Although they call attention to the cytoprotective effect of autophagy on cancer cells in the face of nutrient starvation and toxic effects of radiotherapy and chemotherapy, possibly leading them to a state of dormancy [1], little was known at that time about the role of autophagy on tumor progression and if this could be seen as another hallmark of cancer. Since then, evidence had emerged on the pro-survival role of autophagy in cancer cells. In this review, we first proposed a new hallmark - pro-survival autophagy. Then, we modified two enabling characteristics that play a major role in enabling cells to acquire the hallmarks of cancer and discussed the recent updates, therapeutic and prognostic implications of the eight hallmarks of cancer described by Hanahan et al. in 2011 (Figure 1) [1].

## Review

New hallmark of cancer

Pro-survival Autophagy

Autophagy is an important process in cell death, conservation of protein homeostasis and maintenance of normal organelle function, as it removes damaged structures under cell environment stress conditions. Autophagy can occur in three different pathways: chaperone-mediated autophagy (with the participation of intermediate ligand chaperone proteins like HSP70s), microautophagy (direct engulfment of small cytoplasmic structures by lysosomes), and macroautophagy (where cytoplasmic components are sequestered into the autophagosome and degraded on lysosomes) [3].

Autophagy is essential for oncogenic K-Ras-induced malignant cell transformation in human breast epithelial cells, as the mRNA protein levels of ATG5 and ATG7 (autophagy-specific genes) were increased in cells overexpressing K-Ras and that targeted suppression of these genes inhibited cell growth and tumor formation [4]. Tan and colleagues observed that autophagy was increased in hypoxic regions of tumors in three different human tumor cell lines and hypoxia-induced cell death was more rapid in their autophagy-deficient variants with shRNA knockdown of the genes ATG7 and BECLIN1 [5]. Amid situations of metabolic stress in tumors, autophagy is required to maintain the pool of functioning mitochondria through recycling damaged or nonfunctional intracellular material. This process ensures the supply of nutrients (like amino acids and fatty acids) and ATP, needed for tumor growth [6]. Deficiency in autophagy leads to the accumulation of abnormal mitochondria, lack of ATP, and key tricarboxylic acid (TCA)-cycle intermediates, resulting in mitochondrial dysfunction. Mitochondrial dysfunction leads to the generation of toxic reactive oxygen species (ROS) and mitochondrial damage [7] and is an important process in pancreatic tumor growth [8], as well as liver tumor formation in ATG5 and ATG7 deficient mice [9].

Although not totally clear, some studies suggest a positive correlation between autophagy and epithelial-mesenchymal transition (EMT), which is needed in cancer progression and metastasis, probably via p62 (an autophagy adaptor protein) and tumor growth factor β (TGF-β), which is the most important regulator of EMT in human cancers [10]. Knockdown of BECLIN1, an important autophagy activator, was shown to suppress EMT in colon cancer [11], and its activation via phosphorylation by ULK2 promotes EMT in lung cancer cells [12].

Because autophagy facilitates cancer cells' survival in an environment with hypoxia and metabolic stress, it reduces tumor necrosis and consequently the infiltration of macrophages in the primary tumor, that is a required step for metastasis [10]. Also, autophagy provides tumor cells the ability to avoid anoikis, a process of detachment-induced programmed cell death, resulting from loss of, or inappropriate cell adhesion from the extracellular matrix (ECM) via RNA-like endoplasmic reticulum kinase (PERK), that promotes autophagy and ROS detoxication in mammary epithelial cells [13], upregulation of BNIP3 mediated by ERK/HIF-1α pathway induced autophagy by suppressing mTOR/S6K1 [14] and deficiency of miRNA-30a, a tumor suppressor that targets Beclin-1 and ATG5 in hepatocellular carcinoma [15]. Anoikis resistance also occurs via the regulation of SDCBP/MDA-9/Syntenin in gliomas [16].

Cancer stem cells (CSCs) that play an important role in tumor recurrence and resistance to anti-neoplastic treatment strategies, seem to have a role in tumor maintenance and function related to autophagy. Some autophagy markers like ATG5, ATG12, and LC3B were found to be overexpressed in dormant stem cell-like breast cancer cells [17] and BECLIN1 staining areas were found to be surrounded by actively proliferating cells [18]. In pancreatic cancer, HIF-1α and autophagy regulate the balance between CSCs and non-CSCs [19]. A subset of CSCs showed resistance to chemotherapy and was associated with a high autophagy activity [20].

Autophagy, as an important survival mechanism against various cellular stresses, can induce resistance to various anti-cancer therapeutic agents (cytotoxic chemotherapy, radiotherapy, molecular target agents and antiangiogenic agents) by reducing ROS damage, blocking apoptosis, and maintaining the CSC pool [10]. Some therapeutic agents like imatinib and paclitaxel have already known mechanisms of resistance related to autophagy [21,22].

The better comprehension of pro-survival autophagy leads to studies assessing classes of therapeutic anti-cancer targets in this field, most of them with preclinical evidence, but some are still ongoing clinical trials. One of them are class III isoform of phosphoinositide 3 kinase (PI3K), that induces autophagy by generating PI3-phosphate, needed for the formation of autophagosome membrane [23]. Wortmannin, a covalent irreversible binding inhibitor of PI3K have demonstrated enhanced response when associated to cisplatin in urothelial cancer [24], and spautin-1, which blocks autophagy by deubiquitinating Beclin-1, improved imatinib-induced apoptosis in chronic myeloid leukemia [25]. The ULK1 inhibitor SBI-0206965 attenuated cell survival in non-small cell lung cancer tumor cells [26], reduced tumor growth and metastasis in neuroblastoma cells [27] and exhibits a potential anticancer effect against clear cell renal carcinoma [28]. ATG4 inhibitors NSC185058 and Ticonazole, respectively, exhibit anti-tumor effect in osteosarcoma tumor models in vivo [29] and enhance the cytotoxicity of chemotherapeutic drugs, suppressing tumor viability [30]. Hydroxychloroquine (HCQ) attenuates lysosomal acidification, resulting in obstruction of the activity of the lysosomal degradative enzyme and inhibiting autophagy, but at a high concentration with greater cytotoxicity [31]. Lys05, a potent autophagy inhibitor derivative of HCQ demonstrated anti-tumor activity [32]. Bafilomycin-A1 specifically obstructs V-ATPases and blocks autophagy flux by inhibiting lysosomal acidification, facilitating cell cycle arrest and caspase-dependent apoptotic cell death in colon cancer [33].

The importance of pro-survival autophagy is well established in tumorigenesis, as it is related with a lot of events in cancer progression and critical for some of them in various types of cancers. Besides that, autophagy is a potential anti-cancer therapeutic target. For this reason, autophagy is a good candidate for a new hallmark of cancer.

Enabling characteristics

Tumor Promoting Microenvironment and Macroenvironment

Hanahan et al included tumor-promoting inflammation as one of the enabling characteristics of cancer [1]. Most tumors trigger a tumor-promoting inflammatory response [34] which is recognized as the seventh hallmark of cancer [35]. Inflammation plays a major role in every step of cancer development [34,36].

Tumor initiation: The inflammatory microenvironment increases mutations and genetic instability either by producing ROS and reactive nitrogen intermediates or via cytokines that stimulate ROS [34,36]. Inflammatory cytokines express activation-induced cytidine deaminase and inhibit p53 thereby increasing genetic instability and mutations [34]. Inflammation promotes stem cell expansion [34].

Tumor promotion: Inflammatory response induces genes promoting cell proliferation and survival [34]. Tumor associated macrophages in response to hypoxia produce vascular endothelial growth factor (VEGF) promoting neo-angiogenesis [34,36].

Tumor metastasis and invasion: Inflammatory myeloid cells and cancer cells produce tumor growth factor β that helps in EMT and metastasis [34]. Inflammatory cells produce proteases to facilitate proteolysis, that is required for invasion [34,36]. Survival of metastatic seeds is assisted by cytokines [34]. Inflammation also upregulates adhesion molecules that facilitate the attachment of metastatic cells in target organs [34].

The role of several inflammatory cytokines as prognostic markers is being studied. Higher levels of IL1 β, IL1Ra, IL18, and IL1α in breast cancer tissues and significantly higher IL1 β levels in stage II, III or IV breast cancers were reported [37]. Higher levels of IL-17+ and IL-22+ T lymphocytes are associated with the progression of basal-cell and squamous cell skin cancers [38]. Targeting these inflammatory cytokines might have a potential therapeutic effect in these cancers.

The inflammatory cells acquire pro Vs antitumor capabilities depending on the interaction with TME and the macroenvironment. In addition to tumor-promoting inflammation, there are several studies in the last decade highlighting the bidirectional interaction between a cancer cell and microenvironment as well as macroenvironment that is key for a cancer cell to develop, survive, progress, and invade. Tumor exosomes play a major role in communication with TME and the macroenvironment to promote tumor growth and metastasis. Tumor exosomes modify stroma and immune cells creating a metastatic niche supporting the seeding of tumor cells [39].

Tumor macroenvironment also referred to as tumor organismal environment includes metabolic, endocrine, lymphatic, hematopoietic, immunologic, microbiotic and neurogenic environments [40]. Metabolic imbalance in the macroenvironment seen in two major metabolic disorders, diabetes and obesity, is shown to be associated with promoting tumor development [40]. Hormonal changes in the endocrine macroenvironment impact tumor growth and tumor-promoting inflammation [2]. Tumors also control the endocrine environment via paraneoplastic syndromes [41]. The lymphatic environment helps tumors communicate with systemic circulation [40]. Hematopoietic and immunologic environments influence bone marrow thereby facilitating tumors to suppress antitumor immunity [40]. Recent studies demonstrated the role of gut microbiota in inflammation-promoting tumorigenesis. Gut microbiota promotes tumor progression via the production of certain inflammatory cytokines such as TNFα, IL-6, and IL-17 [2]. Gut microbiota dysbiosis was reportedly related not only to local inflammation-promoting tumor development but also distant cancer such as hepatocellular carcinoma and breast carcinoma and liver metastases in lymphoma [40]. Further studies are required to investigate the effect of modifying gut microbiota dysbiosis in promoting antitumor immunity. The neurogenic environment is particularly gaining interest recently. Zhao et al demonstrated that denervation suppresses gastric tumorigenesis [42]. Furthermore, the concept that adrenergic nerves drive tumor angiogenesis was recently described [43]. Tumor exosomes are reported to control tumor-promoting neurogenesis and glutamate released from local neurites is reported to facilitate breast to brain metastases [40]. Sympathetic and parasympathetic signaling showed a role in the growth of prostate cancer [40].

Cancer Epigenetics, Genome Instability and Mutation

Epigenetics is defined as heritable modifications in gene expression induced via changes in chromatin structure barring adjustments of DNA sequence [44,45]. Accumulating evidence indicates that, without genetic alterations, epigenetic mechanisms are implicated in the acquisition of malignant phenotype [46]. These genetic and epigenetic alterations interact at all levels of the development of most cancers, working collectively to promote cancer progression [47]. The genetic foundation of cancer is widely accepted; however, recent research proposes that epigenetic changes may also be the key initiating events in some types of cancer [48].

Epigenetic mechanisms that regulate chromatin structure can be divided into four essential categories - DNA methylation, covalent histone modifications, non-covalent mechanisms such as the incorporation of histone variants and nucleosome remodeling and non-coding RNAs together with microRNAs (miRNAs) [49,50].

The altered epigenetics of most cancers cells suggests that epigenetic therapies should have a fundamental clinical impact [47]. The main question remains if we can use our understanding of the epigenetic regulators to find so-called synthetic vulnerabilities that would give us new therapeutic possibilities in the treatment of cancer?

In 2011, Hanahan and Weinberg added genome instability into their list of fundamental characteristics of cancer, particularly as an enabling characteristic [1]. The result of the failure of crucial teams of proteins that protect the DNA of the genome from being mutationally corrupted, rearranged, and re-programmed is ‘oncogenic’ mutations that convey on cancer cells various hallmark capabilities. Very recently, Pan-cancer Analysis of Whole Genomes (PCAWG), an international collaboration to identify common patterns of mutation, published an analysis of 2,658 whole cancer genomes across 38 tumor types (https://www.nature.com/collections/afdejfafdb/Feb2020). One of the major studies of this project shows that each tumor had four or five driver mutation on average and at least one driver mutation was found in about 95% of the tumor samples compared with just 67% with exome sequencing [51]. Cancer develops through a process of somatic evolution [52]. Early oncogenesis is characterized by mutations in a constrained set of driver genes and specific copy number gains [52]. However, the mutational spectrum changes significantly throughout tumor evolution in 40% of samples. New patterns of mutations were reported that result from environmental exposures such as tobacco smoke [53]. Endogenous sources of mutation and epigenomic features promote genomic instability during cancer evolution [54]. Recent work has shown that even in repair-sufficient cells, endogenous and oncogenic stress can occasionally overwhelm the normal genome maintenance pathway [54].

Solid tumors of epithelial origin with extreme levels of genomic instability are associated with a potentially better prognosis compared with intermediate level [55]. Also, cancers with extreme levels of genomic instability may be teetering on the brink of a threshold where so much of their genome is adversely altered that cells rarely replicate successfully [5] these cancers are more immunogenic than other cancers with a less extensive burden of genetic aberrations. Pua et al. reviewed studies that established a link between inflammation and the pathophysiology of cancer, and this stems largely from the genomic instability that results from inflammatory cytokines and signaling [56]. Inflammation leads to suppression of cell cycle arrest and apoptosis, further allowing the proliferation of mutant cells.

Recent fast progress in CRISP/Cas9 base editing technology has made it technically highly feasible to generate site specific nucleotide substitutions of DNA by manipulating highly intricate DNA repair pathway [57].

An update to 2011 hallmarks of cancer

Sustaining Proliferative Signaling

One primary hallmark described by Hanahan et al is the cell's ability to become auto-sufficient and enable signals to sustain a continuous proliferative pathway [1]. Some messages can be extracellular growth signals, which bind to transmembrane receptors or transcellular and intracellular stimuli [1]. The mitogenic signals and regulation are complex, and the activity seems to be controlled from one cell to the others as paracrine signaling [1]. In cancer cells, this process is better comprehended, and mutant cells obtain the ability to replicate in many ways [58-61].

The mitogenic stimulus can be generated by an upstream receptor pathway or by downstream and intracellular circuits.

Upstream circuits: Tumor cells may induce the surrounding cells to support their growth with various growth factors [62,63]. The cancer cell may have the ability to increment the number of displayed transmembrane receptors. It will make them hyper-responsive to GF ligands and increase the proliferative rate. These receptors usually have intracellular tyrosine kinase actions. For example, EGF-R/erbB in Breast, stomach, and brain tumors. A structural modification can make these ligand-independent receptors easily firing. For example, some truncated versions of the EFG missing part of their cytoplasmic domain fire continuously [61].

Downstream cytoplasmic pathways may be GF independent and result in continuous activation of proliferation.

a. Somatic mutations trigger more downstream circuits: As shown by Davies and Samuels, 40% of melanomas have some mutations in B-Raf protein result in continuous signaling generated by the activation of Raf to MAP- kinase (mitogen-activated protein). Mutations in phosphoinositide 3-Kinase (PI3-kinase) isoforms may hyper-stimulate the signaling pathway [64,65].

b. Disturbances of Negative-Feedback mechanisms that attenuate proliferative signaling: Antiproliferative signals control and maintain cell homeostasis by inhibition of proliferation [66-69]. Failure in a negative feedback mechanism will allow the cell to replicate indefinitely. For example, the Ras oncoprotein: does not increase the activity of the signal. Instead, it compromises an enzyme action, the Ras GTPase, which is responsible for transitory signal in the Ras pathway. The mutation compromises the negative feedback and results in continuous activation and signal transduction. The PTEN phosphatase counter-acts PI3K Kinase. If PTEN presents a loss-of-function mutation, it may result in the amplification of PI3K signaling, leading to tumorigenesis. mTOR kinase results in negative feedback and inhibition of PI3K signal. The repression of mTOR will result in increased PI3K activity and its effector Akt/PKB, causing dampening in the antimitotic effects of mTOR inhibition [70,71].

These mechanisms of turning off the negative control of cell proliferation probably contribute to drug resistance. The excessive proliferative stimulus may result in cell senescence or apoptosis [72-74]. This contradictory behavior may reflect intrinsic cellular protection, which attempts to eliminate cells presenting inappropriate proliferation signals. Some cancer cells probably adjust their tolerance to abnormal oncogenic protein levels by deactivating their apoptosis or senescence-inducing pathways.

Evading Growth Suppressors

Cancer cell needs to avoid antigrowth signals to thrive [75]. Growth suppression resides centrally in pRb and p53, which operate as part of a large network that is inter-ligated in a way that favors redundancy and more efficient control. Tumor cells escape these antiproliferative control by loss of function of these genes or alterations in response to their close regulators as TGFβ or immediate downstream targets of its action, as CDK4 and its inhibitory proteins [75] and contact inhibition of cell growth, like NF2 / Merlin and LKB1 epithelial polarity protein, that when disrupted can also facilitate uncontrolled proliferation as seen in cancer tissues [1].

Several other mechanisms, directly or not linked to pRb and p53 pathways, positively or negatively related to antiproliferative control, emerged in the last years and confirm the evasion of growth suppressors as an actual hallmark of cancer, helping to understand widely the complexity of this system, which can be a potential therapeutic target in many types of cancers.

Alternative Reading Frame (ARF)

ARF is a tumor suppressor protein encoded in the INK4b/ARF/INK4a gene locus located on chromosome 9p21 in humans that activate p53 in response to oncogenic signals, such as c- MYC [76]. Although transcriptional regulation of ARF has been known since the late 1990s, most of its post-translational regulation have emerged after 2004, that this protein could be ubiquitinated and degraded via proteasomal degradation [77] and the enzymes involved in this process like ULF, SIVA1 and MKRN1 were discovered only after 2010 [78-80]. Besides that, ARF can also suffer lysosomal degradation mediated by CHIP and HSP90 [81]. Inhibition of HSP90 by geldanamycin can induce cell growth retardation and cellular senescence in human normal cells and mouse embryonic fibroblasts [81]. De-ubiquitination of ARF by USP10, which results in stabilization of ARF and promotes cellular senescence [82].

The low expression of ARF mRNA is frequently observed in human cancers and is usually caused by hyper-methylation on the CpG island of the ARF promoter or deletion of the genetic region and has been described in breast, bladder, colon, liver, gastric, lung, oral, prostate and brain cancers and has emerged as a predictor of poor prognosis in breast, head and neck, colon and bladder carcinomas [82].

Galectins

Glycosylation changes had emerged as an important process in cancer progression and among the glucan-binding proteins that deciphers the information encrypted by the glycoma, galectins had great importance [83]. Gal7 showed to be a proapoptotic Galectin induced by TP53 in colorectal and urothelial cancer [84,85]. Gal7 expression can be silenced by methylation of CpG islands in the LGALS7 gene and hypermethylation at a region of the exon2 that is predicted to be a TP53-binding [86]. Nevertheless, the overexpression of Gal7 had a tumor-promoting behavior in thymic lymphoma and breast cancer [87,88], a paradoxical effect that could be explained by the observation that both NF-κB-binding and TP53 transcription factors can control its expression. In breast cancer cell lines, WT and mutant TP53 increased NF-κB activity and up-regulated Gal7 expression. Conversely, in a p53-null cell line, with high NF-κB activity, Gal7 undetectable, suggesting NF-κB-TP53 complex to be required to the LGALS7 promoter activation [89]. Reciprocal regulation between Gal7 and TP53 was also proposed when observed that Gal7 was able to impede TP53 translocation to the nucleus [90]. Another galectin, Gal3, was shown to be repressed by TP53, with increased expression of Gal3 been observed in p-53 mutant tumors [91]. In human prostate cancer cells, knockdown of Gal3 promoted a cell cycle arrest at the G1 phase, up-regulation of nuclear p21, and hypophosphorylation of Rb [92].

Melatonin

Melatonin was found to induce phosphorylation of p53 at Ser-15 causing proliferation inhibition and prevention of DNA damage accumulation [93]. The treatment with melatonin resulted in increased p53 expression in breast cancer and prostate cancer cells and expression of BRCA 1 and BRCA 2 in breast cancer cells [94,95].

Micro-RNAs (miRNAs)

Many critical cell proliferation pathways involve miRNAs, dysregulation of which is responsible for evading growth suppressors and sustaining proliferative signaling in cancer cells. Some of these miRNAs interfere on E2F proteins expression, that are cell cycle regulators of cell proliferation, as miR-17-92 that inhibits E2F1 translation through breaking the positive feedback between c-Myc and E2F1 [96] and was found to regulate E2F2 and E2F3 translation [97] in a feedback system that promotes normal cell proliferation. The overexpression of miR-17-92 in some tumors like B-cell lymphomas can disrupt this feedback loop and lead to unbalanced cell proliferation [98].

Other microRNAs negatively regulate CDK inhibitors, being needed to the cell cycle progression and cell proliferation, as miR-221/222, which has been identified to directly target p27Kip1 in glioblastoma cells [99] and that its overexpression can be found in a variety of human tumors [100], confirming the oncogenic role of this pathway. MicroRNAs can also directly regulate the expression of CDK and cyclins, as an example of miRNA-545, that promotes cell-cycle arrest in lung cancer cells by repressing the expression of cyclin D1 and CDK4 [101].

Long Noncoding RNAs (lncRNAs)

There are more than 10,000 lncRNAs in the human genome and they are implicated in almost all hallmarks of cancer, including acting as regulators of tumor suppressor genes and molecules that regulate the cell cycle [102]. The lncRNA HULC regulates the expression of P18 protein (an inhibitor of CDK4 and CDK6) and its high expression is correlated with hepatocellular carcinoma. H19 derives miR-675, which negatively regulates RB and p53 [103]. LINCRNA-P21 inhibits CDK2 and is found to be downregulated in hepatocellular carcinoma [104], the same way that PTENP1 downregulation promotes malignant behavior in head and neck squamous cell carcinoma [105]. HEIH negatively regulates the expression of CDK inhibitors P15, P16, P21 and P57, via EZH2 [106] and ANRIL positively regulates proliferation by recruitment of polycomb repression complex 2 to the INK4 locus and represses the transcription of P15 [107].

Resisting Cell Death

Cancer formation involves different changes in the genome [108] and a series of genetic alterations and failure of intracellular checkpoints [109]. Defective apoptosis is a hallmark of cancer [75] and understanding its mechanisms can help in designing efficient therapeutic strategies.

Apoptosis pathways are classified into two types: extrinsic (receptor-mediated) and intrinsic (mitochondria-mediated). These pathways may be linked, molecules involved in one can affect the other one, and they can also be involved in other cell processes [110]. The extrinsic pathway requires the successful interaction of specific receptors found on cell membranes and their respective ligands. The intrinsic pathway may be activated by different stimuli, like radiation, free radicals, viral infections, and serum/growth factor withdrawal [110]. The triggers in the mitochondria-mediated pathway change the inner mitochondrial membrane permeability resulting in the loss of the mitochondrial transmembrane potential, the release of pro-apoptotic proteins and the arrest of the bioenergetic function of the organelle [110]. This pathway does not include special receptors; thus, its exploitation may be a key to successful cancer therapy [110].

The regulation of apoptosis is also complex. The Bcl-2 family includes different proteins that influence apoptosis: some of them interact with mitochondrial proteins and prevent them from forming pores, inhibiting the release of apoptogenic factors, others neutralize the anti-apoptotic proteins [111,112]. Interaction between pro-and anti-apoptotic proteins is another way of affecting the survival and death of the cell. Modulation of the regulatory proteins is a major factor contributing to the survival advantages of cancer cells. The inhibitors of apoptosis (IAPs) block cell death by regulating the caspase cascade and may influence both the intrinsic and extrinsic pathways in the cells. The tumor suppressors, like p53, have several action mechanisms, one of which affects mitochondria by p53 physically interacting with Bcl-2 and antagonizing its anti-apoptotic function. ROS also influence cell death signaling. Though ROS can influence the extrinsic death pathway by making the environment conducive for effective interaction of receptors and ligands, the intrinsic pathway is more susceptible to ROS. There are multiple targets for ROS in mitochondria, linked to its primary function of a powerhouse in cells [113].

The main issue with conventional therapies in cancer treatment (surgery, radiation, and chemotherapy) is the evolution of adaptive mechanisms in cancer cells. Adaptive mechanisms may include upregulation of pro-survival proteins, suppression of pro-apoptotic proteins and defects in p53 signaling pathways. Many anticancer agents that target pathways involving deregulation of proteins like p53 lead to resistance, so direct targeting of mitochondria may be a promising strategy in attempts to restore the cells’ ability to die [114-117].

In conclusion, new drug targets can be identified and target selective therapeutic methods could be developed through analysis of apoptotic signaling pathways and apoptosis resistance mechanisms.

Enabling Replicative Immortality

The ability of tumoral cells to achieve replicative immortality, allowing subsequently macroscopic growth, has been widely accepted as a hallmark of cancer [1]. Several studies point out the central role of telomeres maintenance necessary to bypass the natural cell aging process [118,119]. The gradual telomere shortening, which occurs after multiple cell divisions, regulates the life span of the cells and their capacity for replication [120]. When a telomere becomes too short and reaches a critical length, usually after 50 to 60 cell cycles, the cell senescence is induced [119,121]. Subsequently, genes like p53, p21 and Rb/p16 INK4A that are associated with growth cell arrest and apoptosis, are activated. When a short telomere is not recognized by the cell cycle arrest checkpoint, it will undergo further shortening and DNA damage proteins will be activated, leading to homologous recombination or non-homologous end joining of the chromosome, generating thereby aberrant chromosomes. Such a cell with unstable DNA is defined in the crisis phase and normally undergoes apoptosis. This process of telomere shortening inducing senescence or crisis/apoptosis in normal cells is considered a sort of natural tumor-suppressing mechanism.

In carcinogenesis, cells can activate mechanisms of telomere maintenance to overcome the cell senescence or apoptosis caused by telomere shortening. Several mechanisms of telomere maintenance have been identified and include telomerase gene hTERT promoter mutations [122], telomerase reactivation, oncogenes and tumoral suppressor genes mutations, alternative lengthening of telomeres (ALT) - telomerase independent mechanism [123].

According to data, in 85%-90% of cancer cells, the mechanism of telomere stabilization is reached by telomerase activation and only 5%-15% exhibit an ALT pathway [124-126].

Despite the upregulation of telomere reverse transcriptase (TERT) expression via promoter mutation [127-132] and telomerase activation in most malignant cells, telomeres in cancer are shorter than those in normal tissues: as several studies show, 90% of cancer cells contain short telomeres and high levels of telomerase activity. In fact, it seems the shorter the telomere is, the higher the cancer aggressiveness, and the poorer the prognosis [132,133]. For example, 75% of oral carcinomas, 80% of lung cancers, 84% of prostate cancers, 85% of liver cancers, 93% of breast cancers, 94% of neuroblastomas, 95% of colorectal cancers, and 98% of bladder cancers have detectable telomerase activity [134,135]. Some cancer cell lines keep noticeably short telomeres (prostate PC-3, stomach MKN74 and breast HBC-4 cancer cells) and elongate their telomeres by TERT over-expression [136,137]. Patients with glioblastoma with isocitrate dehydrogenase 1 (IDH1) mutation and ALT activation, which is associated with longer telomeres, showed better clinical outcome than those with ALT negative tumor [133,134,138-140]. Interestingly, TERT or even telomerase activity is inversely correlated with telomere length [133,141].

Huang et al. showed that upon mitogen stimulation, not all but only a small subpopulation of T- cells reactivate telomerase and preferentially elongate short telomeres [142]. It is possible that there are various cell subpopulations with long to short telomeres during cancer development [142]. Furthermore, genomic instability elicited by shortened telomeres might be advantageous to cancer evolution [143]. In fact, induction of chromosomal instability via the telomeric DNA damage response followed by end-to-end fusions promotes oncogenic transformation [144,145]. Telomerase activation confers immortality but not neoplastic properties to cancer cells [135,146], given that malignant transformation requires a multi-step process [135,147].

Nevertheless, should the precise moment of telomeres shortening triggering telomerase activation be known, this could then have major effectiveness in stopping the tumor. This remains a challenge for future researchers [126].

Inducing Angiogenesis

Angiogenesis is a physiological process, that determines the formation of new vessels from preexisting ones. Angiogenesis is involved not only in embryonic development but also in damage and recovery. This process is tightly regulated and controlled by different mechanisms. By contrast, in pathological conditions (like cancer) angiogenesis is dysregulated and hyperactivated. The relationship between angiogenesis and cancer was first described in 1968 when several proangiogenic factors were discovered [148,149].

Tumor angiogenesis is a multistep process, and its main generator is hypoxia in tumor cells due to inadequate blood supply. It causes the production of angiogenetic factors such as vascular endothelial growth factor (VEGF), basic fibroblast growth factor (bFGF), angiogenin, transforming growth factor α, TGF-β, tumor necrosis factor (TNF)-α etc. by cancer cells, which bind to endothelial cell receptors of the vessels and initiate above mentioned process. Whenever the endothelial cells are stimulated, the secretion of matrix metalloproteinases (MMPs) is prompted, which causes degradation of the basal membrane. This process allows endothelial cells to invade surrounding tissues and start forming new vessels. In addition, factors such as angiotensin-1, -2, and their receptor Tie-2c are needed for the stabilization of newly formed vessels [150,151].

Angiogenesis plays a key role in cancer and according to many studies level of angiogenetic factors is correlated with tumor aggressiveness and has a strong predictive role [1,152].

Activating Invasion and Metastasis

**Figure 2:**
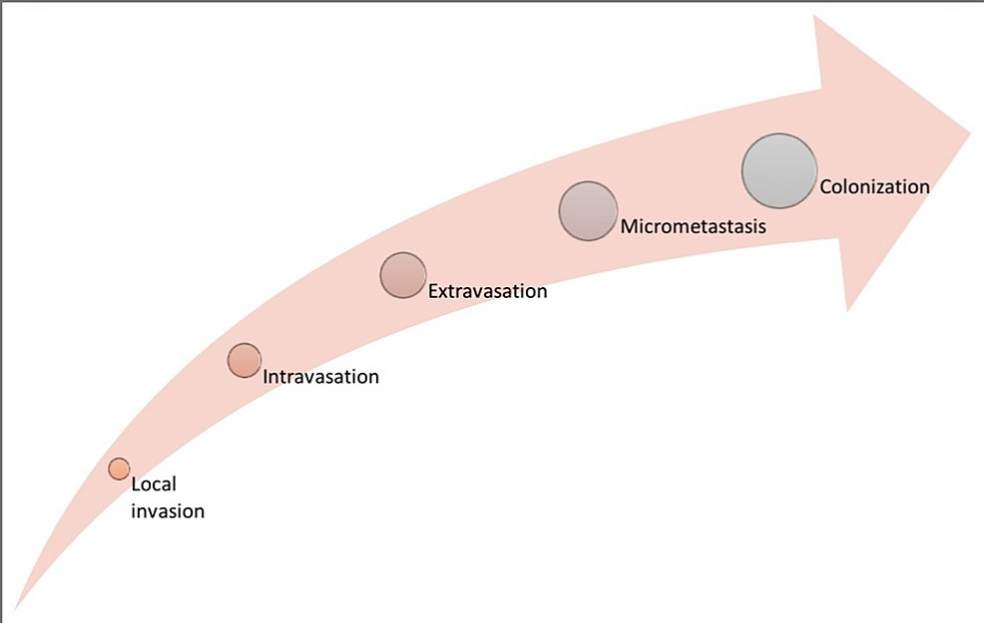
Invasion-Metastasis cascade

Metastasis is a hallmark of cancer and the cause of most cancer-related deaths [1]. It is a multistep process by which tumor cells leave the primary tumor, travel to a distant site, and establish secondary tumors in distant organs (Figure 2) [1,153].

There are a variety of determining factors that govern the flexibility of a primary tumor to metastasize to different organs. These include genetic disorders to growth factors within the environment of the first tumor, the flexibility of tumor cells to detach from neighboring cells, the flexibility of tumor cells to digest the ECM and invade the vasculature. The tumor cells reorganize their cytoskeleton, express adhesion molecules on their surface to acknowledge adhesion sites, and chemotaxis or become migratory and motile resulting in loss of contact inhibition, and ultimately migrate to inappropriate locations giving rise to metastatic dissemination.

In vivo as well as in vitro studies showed that metastatic tumor cells migrate individually [154]. However, in humans, seeding requires the joint action of a cluster of cancer cells moving together, which brings EMT into the picture [155]. EMT program is believed to be a spectrum of transitional stages between the epithelial and mesenchymal phenotypes, in contrast to a progression that includes a binary choice between full-epithelial and full-mesenchymal phenotypes [156].

In recent years, there has been a vital debate on whether EMT features a central role in cancer metastasis and resistance to chemotherapy [157]. Some studies in pancreatic and lung cancers showed that EMT is not essential for metastasis, but it does contribute to chemoresistance. More evidence is required to completely elucidate the role of EMT in cancer progression and the metastatic process [158-160].

Another important thing that researchers are looking at is ECM. ECM is a dynamic and complex system that is composed of a wide spectrum of matrikines and cells that take part in invasion and metastasis [161].

Our deep understanding of the dynamics of this hallmark will help us identify targets for molecular therapies which will halt or possibly reverse cancer growth and metastasis.

Reprogramming of Energy Metabolism

Abnormally high metabolic rates by cancer cells alter anti- tumor immunity by changing TME in the metabolic mechanism of glycolysis or amino acid metabolism. There are studies discussing the crosstalk between energy reprogramming in cancer cells and its association with antitumor immunity, and therefore suggest intervention of cancer metabolic agents provide an add-on benefit to cancer immunotherapy [162].

Glycolysis and Lactate Production

The Warburg effect shows that tumors and cancer cells have increased rates of glucose uptake and lactate production, even in the presence of sufficient oxygen and low rate of oxidative phosphorylation [163]. Metabolic reprogramming in tumor cells causes changes in TME by competing metabolic environment in neighboring T cells, that lead to T cell metabolic exhaustion. Notably, several clinical studies have revealed that aerobic glycolytic activities in humans is negatively correlated with intrinsic antitumor immunity. For example, LDHA (lactate dehydrogenase A)-associated lactic acid accumulation in melanomas inhibits tumor surveillance by T and NK cells, and LDHA mediated lactate production suppresses IFN-γ expression in both tumor-infiltrating and immune evasion murine models [164]. Pyruvate kinase muscle isozyme M2 (PKM2) has a crucial role in sustaining nutrients demands in cancer cell proliferation [165]. Recently, PKM2 has been reported to promote PD-L1 (programmed cell death 1 ligand 1) expression in tumor, and PKM2 activators might synergize with PD-1 (programmed cell death 1)/PD-L1 checkpoint inhibitors to provide suppressive roles to tumor immune escape [166].

Amino Acids Metabolism

Tryptophan (Trp) and arginine (Arg) amino acids are considered to provide key nutrients in TME. Trp attenuates antitumor immunity in primary tumors and the neighboring tumor lymph nodes and Arg catabolism has been linked to suppression of antitumor immunity.

Indoleamine 2,3-dioxygenase 1 (IDO1) enzyme is a rate-limiting enzyme in the metabolism of Trp in the peripheral tissues and IDOI inhibitors inhibit the first step of Trp catabolism [167]. IDO1 is overexpressed in human cancer cells, suppresses effector T cell function, and promotes regulatory T cells [168]. Tumor or stromal cells in malignant lesions catabolizing Trp and/or Arg suppress CD8+ effector T cells and stabilize Treg cells to protect tumor cells [169].

PD-1 ligation and activation impairs metabolic reprogramming, including glycolysis and amino acid metabolism in T cells by inducing the expression of carnitine palmityl transferase 1A (CPT1A), a rate limiting enzyme of the fatty acid oxidation (FAO) pathway, and conversely, CTLA-4 inhibits glycolysis without augmenting FAO [170]. Arginine inhibitor, INCB001158 combined with immune checkpoint inhibitor pembrolizumab, is being studied in advanced or metastatic solid tumors (ClinicalTrials.gov Identifier: NCT02903914). Epacadostat, an IDO1 inhibitor, is being studied in combination with pembrolizumab in patients with metastatic and/or locally advanced sarcoma (NCT03414229). Anti-IDO-1 agent (LY3381916) is also tested in combination with anti- PD-L1 checkpoint antibody (LY3300054) in solid tumors (NCT03343613). Additionally, a phase 2 study is ongoing to evaluate the activity of PD-1 inhibitor, nivolumab alone with and without IDO-inhibitor, BMS-986205, in patients with recurrent or persistent endometrial carcinoma or endometrial carcinosarcoma (NCT04106414). Given great and extensive interests of IDO inhibitors and other metabolic agents, it is expected there will be more clinical studies underway in addressing metabolic intervention in TME to aid on immunotherapy.

Evading Immune Suppression

Evading antitumor immunity plays a major role in tumor progression and survival and must be considered as one of the hallmarks of cancer. Tumor escapes immune destruction by several mechanisms. TGFβ plays a significant role in inhibiting T helper cell differentiation and promoting antitumor immunity [171,172]. Tumor derived factors convert immature myeloid cells into myeloid derived suppressor cells (MDSC) that suppress antitumor immune response [2]. Tumor recruits and educate immune cells such as NK cells, regulatory T cells, dendritic cells, granulocytes, macrophages, and MDSC thereby creating permissive microenvironment [173]. Tumor modulates spleen and bone marrow via exosomes creating permissive macroenvironment [173]. Tumor exosomes not only impair T cell function, but also produce monocytic MDSC that impairs tumor recognition by immune cells [39]. ECM can impair antigen presenting cells and inhibit T-cell activation suppressing T-cell function against tumor [174]. Commensal microbiota is also reported to have a role in impairing antitumor immunity [40].

In this era of immunotherapy, several studies are underway to improve anticancer therapy based on these mechanisms of evading immune suppression. Commensal microbiota was reported to have a role in improving anti-tumor response to immunotherapy and chemotherapy in extraintestinal tumors [2]. Blocking TGFβ signaling could potentially promote antitumor immunity [171], which is proved in advanced colorectal cancer using TGFβ inhibitors [172].

## Conclusions

In conclusion, we summarized the hallmarks of cancer. Pro-survival autophagy is described as a new hallmark of cancer given its important role in tumorigenesis and potentially an anti-cancer therapeutic target. The bidirectional interaction between a cancer cell and microenvironment as well as macroenvironment is key for a cancer cell to develop, survive, progress, and invade. In addition, cancer epigenetics, genome instability and mutation play a major role in enabling cells to acquire the hallmarks of cancer. Despite the advanced understanding, we have so far on hallmarks of cancer, there is much more to uncover in this field of research. Future research in this field is necessary to develop better ways to treat cancer.
